# Investigating Nickel-Induced Neurotoxicity: Associations with Gut Microbiota Dysbiosis and Ferroptosis

**DOI:** 10.3390/antiox14121478

**Published:** 2025-12-09

**Authors:** Yao Shen, Kai Cao, Wenjuan Zhang, Chun Chen, Chang Gao, Jingran Wang, Tian Xin, Cun Li, Shusheng Tang, Xingyao Pei, Daowen Li

**Affiliations:** 1 Tianjin Key Laboratory of Agricultural Animal Breeding and Healthy Husbandry, College of Animal Science and Veterinary Medicine, Tianjin Agricultural University, Jinjing Road No. 22, Xiqing District, Tianjin 300392, China; shenyaotjau@163.com (Y.S.); zhangwenjuan@tjau.edu.cn (W.Z.); chenchun110212@163.com (C.C.); 18611382529@163.com (C.G.); wjrrr0929@163.com (J.W.); 13682107136@163.com (T.X.); hhlicun@163.com (C.L.); 2 Department of Health Service, Logistics University of People’s Armed Police Force, Tianjin 300309, China; chuckcao325@gmail.com; 3 National Key Laboratory of Veterinary Public Health and Safety, College of Veterinary Medicine, China Agricultural University, Yuanmingyuan West Road No. 2, Haidian District, Beijing 100193, China; tssfj@cau.edu.cn; 4 State Key Laboratory of Medicinal Chemical Biology and Tianjin Key Laboratory of Molecular Drug Research, College of Pharmacy, Nankai University, Haihe Education Park, Tongyan Road No. 38, Tianjin 300353, China

**Keywords:** nickel sulfate, neurotoxicity, ferroptosis, autophagy, *TFEB*

## Abstract

Nickel is a pervasive heavy metal with the potential for multi-route exposure, raising significant concerns regarding systemic toxicity. Although Ni^2+^ has been implicated in nickel sulfate NiSO_4_-induced neurotoxicity, its underlying mechanisms remain incompletely elucidated. The present study investigates the role of NiSO_4_-induced ferroptosis as a potential contributor to neurotoxicity. C57BL/6 mice were administered NiSO_4_ daily via oral gavage at doses of 50, 100, and 200 mg/kg over 28 days. Neurobehavioral assessments, histopathological examination, transmission electron microscopy, and molecular profiling were conducted to evaluate brain injury and ferroptotic activity. Gut microbiota composition and intestinal barrier integrity were systematically evaluated. In vitro, HT22 cells were subjected to NiSO_4_ treatment, followed by integrative transcriptomic analysis complemented by pharmacological and genetic manipulation to delineate the contributions of ferroptosis and autophagy. The results demonstrated that NiSO_4_ exposure inhibited body weight gain, elicited depression-like behaviors, and initiated ferroptosis, evidenced by ultrastructural mitochondrial damage and dysregulated expression of glutathione peroxidase 4/acyl-CoA synthetase long chain family member 4 (*GPX4*/*ACSL4*). Furthermore, NiSO_4_ caused gut microbiota dysbiosis and compromised the intestinal barrier, which was correlated with the induction of ferroptosis in neuronal cells of the brain. In HT22 cells, NiSO_4_ elicited dose-dependent cytotoxicity and lactate dehydrogenase (LDH) release. KEGG pathway enrichment analysis further revealed that NiSO_4_ treatment significantly upregulated pathways associated with ferroptosis, autophagy, and lysosomal function. Moreover, both ferrostatin-1 and rapamycin attenuated NiSO_4_-induced cytotoxicity and ferroptosis, indicating that autophagy serves a protective function against ferroptotic cell death. Additionally, overexpression of Transcription Factor EB (*TFEB*) attenuated NiSO_4_-induced ferroptosis by downregulating *ACSL4*, and upregulating *GPX4*, implicating the autophagy–lysosome pathway in the protective regulation of this cell death process. In summary, our findings indicated that NiSO_4_-induced neurotoxicity was strongly associated with gut microbiota dysbiosis and coincided with ferroptosis in the brain, while stimulation of the autophagy–lysosome pathway conferred neuroprotective effects via modulating *TFEB*-dependent anti-ferroptotic mechanisms. These findings offer novel insights for risk assessment and therapeutic strategies of nickel-related neurotoxicity.

## 1. Introduction

Nickel, a pivotal transition metal, is extensively utilized across a wide range of industrial applications due to its high ductility, strong oxidation and corrosion resistance, and economic viability. However, the production and processing of nickel can generate nickel-containing waste streams, such as those originating from batteries, catalysts, wastewater, and electrolyte discharges, that may contaminate soil, water, and air, creating environmental hazards and posing risks to human health [1]. Human exposure to nickel is associated with a spectrum of adverse health effects, including allergic reactions, cardiovascular and renal diseases, pulmonary fibrosis, and cancers of the lung and nasal cavity. While the precise molecular mechanisms of nickel-induced toxicity remain incompletely elucidated, accumulating evidence indicates that its toxicological impact is mediated, at least in part, through disruption of mitochondrial homeostasis and induction of oxidative stress [2]. One of the most recognized deleterious effects of nickel, lipid peroxidation (LPO) is driven by oxidative stress. Nickel has been demonstrated to modulate the Fe-H_2_O_2_-mediated LPO process. Research in male Wistar rats exposed to NiCl_2_ has revealed a state of profound oxidative stress in brain, characterized by depleted glutathione (GSH) levels, concomitant reduction in the activities of antioxidant enzymes, and a consequent accumulation of H_2_O_2_, as well as elevated LPO [3]. Excessive nickel intake exhibited neurotoxicity and might promote neurodegeneration. It found that heavy metal-induced neurodegeneration was mediated by the disruption of astrocyte homeostasis, which further critically compromised the neuroprotection in neurotransmitter recycling, redox balance, and metabolic support [4]. In an observational study of employees consuming nickel-contaminated water, the primary neurological symptoms of nickel exposure were embodied in dizziness, fatigue, and headaches [5].

Ferroptosis is acknowledged as a distinct iron-dependent cell death pathway, distinguished by specific features such as disruption in iron homeostasis, the reduced ability to mitigate oxidative stress, and obvious lipid peroxidation. Morphologically, ferroptosis is characterized by cellular swelling, electron-dense mitochondria, outer membrane rupture, and loss of cristae [6]. As a stress response, dysregulated ferroptosis has been verified to contribute to the pathogenesis of diverse conditions, including neurodegenerative disorders, cancers, inflammatory diseases affecting various organs, ischemia–reperfusion injuries, and brain injuries [7]. Specifically, the mechanisms underlying metal-induced ferroptosis involve oxidative stress, lipid peroxidation, ferritinophagy, and mitochondrial stress [8]. Studies have demonstrated that NiCl_2_ could induce mitochondrial damage and trigger hepatic ferroptosis [9]. Additionally, ferroptosis has been implicated in nickel-induced neurotoxicity in zebrafish [10]. Nevertheless, the precise mechanisms underlying nickel-induced neural damage in the brain remain poorly understood.

Autophagy serves as a primary cellular mechanism that facilitates the delivery of diverse cargo to lysosomes for degradation and recycling. It is widely recognized as a critical cytoprotective process in pathogenesis of numerous diseases [11]. Emerging evidence suggested that ferroptosis represented a form of regulated cell death associated with autophagy [12]. In this context, autophagy directly engaged Nuclear Receptor Coactivator 4 (*NCOA4*) to bind the Ferritin Heavy Chain 1 (*FTH1*), forming a ferritin complex, which was subsequently targeted to lysosomes for ferritinophagy [13]. It also demonstrated that the inhibition of autophagy could result in the activation of ferroptosis, and the crosstalk between autophagy and ferroptosis has been observed in atherosclerosis [14]. Further investigations have elucidated that ferritinophagy contributes to the ferroptosis of vascular endothelial cells induced by NiSO_4_ nanoparticles [15]. Moreover, NiCl_2_ has been shown to induce ferroptosis in mouse kidneys and TCMK-1 cells, wherein autophagy significantly contributes by degrading ferritin and facilitating iron accumulation [16].

The homeostasis of the gut microbiota exerts a profound influence on host health, impacting not only the gastrointestinal environment but also distant organs, including the brain. The bidirectional communication of the gut–brain axis is critically mediated by the gut microbiome in regulating the metabolism of host, immune system, and vascular system. The gut microbiota transmits signals to the brain via the vagus nerve, thereby modulating central nervous system activity in a bidirectional manner [17]. It has shown that nickel oxide nanoparticles could cause gut dysbiosis and reduce probiotic abundance by disrupting pivotal metabolic pathways [18]. Furthermore, previous research has demonstrated that oral administration of nickel could alter gut microbiota composition in mice [19]. However, the relationship between nickel-induced gut microbiota alterations and brain injury, as well as its potential link to ferroptosis, remains incompletely characterized. In this study, the toxic mechanisms of NiSO_4_ were investigated, and the role of NiSO_4_-induced ferroptosis in neurotoxicity through both in vivo and in vitro analyses was also accomplished.

## 2. Materials and Methods

### 2.1. Chemicals and Reagents

Nickel sulfate heptahydrate (NiSO_4_·7H_2_O, 99.9% purity) were obtained from Sigma (St. Louis, MO, USA). DMEM high glucose medium and fetal bovine serum were procured from Gibco (Grand Island, NY, USA). Additional cell culture reagents, including the LDH enzyme-linked immunosorbent assay detection kit, the CCK-8 cell proliferation and cytotoxicity detection kit, and the oxidative stress detection kit, were acquired from SolarBio Science & Technology Co. (Beijing, China). The endotoxin detection kit based on the limulus amebocyte lysate chromogenic method was acquired from Beyotime Biotechnology (Shanghai, China). The JC-1 staining kit was sourced from AMRESCO Inc. (Solon, OH, USA), while the fluorescent probe RhoNox-1 for divalent iron ions was purchased from MedChemExpress. NiSO_4_·7H_2_O was dissolved in sterile, ultrapure water for both in vivo gavage and in vitro treatments. Other unspecified reagents were of analytical grade.

### 2.2. Studies in Animals

Adult male C57BL/6 mice (6–8 weeks old, 18–22 g) were sourced from River of Life Animal Technology Co., Ltd. (Beijing, China). All animal experiments were conducted in accordance with protocols approved by the Institutional Animal Care and Use Committee of Tianjin Agricultural University (Approval No. 2024LLSC47 and approval date: 8 September 2024). Mice were maintained under standard housing conditions (12 h light/dark cycle, 22 °C, 50% humidity) with free access to food and water. A total of 24 mice were randomly assigned to four groups: a control group and three experimental groups receiving NiSO_4_ at concentration of 50 mg/kg, 100 mg/kg, and 200 mg/kg, respectively, with six mice per group (n = 6). Mice in the control group were administered distilled water via gavage, while the NiSO_4_ groups were administered NiSO_4_ at the specified doses for 28 consecutive days. The selected doses of NiSO_4_ were based on preliminary experiments and align with established toxicological models, simulating subacute, high-level exposure relevant to scenarios such as feed contamination. These doses, consistent with prior mechanistic studies, enabled clear dose–response assessment without excessive mortality [20,21,22]. Following induction of deep level anesthesia with a lethal dose of pentobarbital sodium (i.p.), blood was collected via terminal cardiac puncture immediately upon confirmation of unconsciousness. Death was confirmed immediately following the procedure by cervical dislocation. Serum samples were collected for the quantification of lipopolysaccharide (LPS) using kits from Nanjing Jiancheng Bioengineering Institute. The brains of the mice were sectioned, with portions allocated for homogenization and others reserved for histopathological analysis.

### 2.3. ELISA for Inflammatory Cytokines

Pro-inflammatory cytokine levels were assessed by ELISA. Serum was isolated from whole blood by centrifugation at 3000 rpm for 15 min at 4 °C. Brain tissues were homogenized in ice-cold PBS containing protease inhibitors using a mechanical homogenizer at a 1:9 (*w*/*v*) tissue-to-buffer ratio. The homogenates were then centrifuged at 12,000 rpm for 15 min at 4 °C, and the supernatants were collected for analysis. Protein concentrations in the brain homogenates were determined using a BCA assay kit (SolarBio, Beijing, China) according to the manufacturer’s instructions. All cytokine levels in brain homogenates were normalized to their respective total protein concentrations. Subsequently, IL-1β, TNF-α, and IL-6 in both serum and brain homogenates were quantified using respective commercial ELISA kits (Nanjing Jiancheng Bioengineering Institute, Nanjing, China) according to the manufacturer’s protocols. All samples were measured at 450 nm using a microplate reader (BioTek Instruments, Inc., Winooski, VT, USA).

### 2.4. Mouse Behavior Analysis

Twenty-four hours after a 28-day intragastric treatment, mice underwent the forced swim test (FST) and tail suspension test (TST). For the FST, mice were placed in 25 cm high, 15 cm diameter glass cylinders filled with water at 25 ± 2 °C. Behavior was recorded with a ceiling camera and analyzed with Smart 3.0 software. The FST spanned two days: on the first day, mice swam for 8 min to induce despair, were dried, and returned to their cages. The next day, they swam for 8 min. Behavior was scored based on immobility, swimming, and climbing. Water was changed after each trial to avoid pheromonal effects. The TST setup included a 40 cm cubic opaque PVC chamber with a metal bar 15 cm from the top. A Logitech C920 camera, placed 50 cm away, recorded the trials. EthoVision XT 15.0 software tracked behavior. Mice were suspended by the last 1 cm of their tails using 3M™ Micropore™ tape at a 30° angle. Each mouse was tested for 8 min in 60 lux lighting, with immobility defined as no movement except for breathing. Immobility was recorded during the last 6 min. The chamber was cleaned with 70% ethanol between trials to avoid scent contamination.

### 2.5. Histopathological Analysis

The brains and small intestines were preserved in 4% paraformaldehyde at room temperature for a duration exceeding 48 h. After fixation, brain and intestinal tissues were rinsed, dehydrated with ethanol, embedded in paraffin, and prepared for hematoxylin-eosin (HE) staining. The paraffin sections were heated at 60 °C for 30 min, dewaxed, rehydrated, and immersed in water. Sections were stained with hematoxylin for 10 min, rinsed in running water, differentiated in ethanol, and rinsed again. The sections were then immersed in 95% and 100% ethanol for 30 s each, cleared in xylene for 5 min, and mounted with neutral gum. For Periodic acid–Schiff (PAS) staining, tissue sections were initially dewaxed and rehydrated to water, followed by a rinse in water for 2 min. This section was then oxidized in a periodic acid solution for 10 min and subsequently rinsed with distilled water, with any excess water removed by gentle shaking. Staining was performed using Schiff reagent for a duration of 10 min, after which the sections were subjected to rinsing under a stream of tap water. Nuclear staining was performed using a nuclear staining solution for 2 to 4 min. This section was then washed under running water for 5 min to achieve a blue hue, with excess water removed by shaking. Finally, the sections underwent routine dehydration, clearing, and were mounted using neutral gum.

### 2.6. Immunofluorescence of Tissues

The sections were impregnated with wax and embedded using an embedding machine. After cooling, serial sections of 4 μm in thickness were obtained using a microtome, mounted on glass slides, and dried at 60 °C for 30 min. Dewaxing was performed in xylene for 10 min, followed by immersion in an ethanol concentration gradient of 100%, 95%, 90%, 80%, 70%, 50%, and 0%, for 5 min each. After allowing for natural cooling, the sections were transferred to a shaker containing PBS and rinsed three times. The tissues were then covered with 3% BSA for 30 min. The sections were incubated overnight at 4 °C with the Mucin 2 (MUC2) primary antibody, followed by three PBS washes and a 1 h incubation with the fluorescent secondary antibody in the dark. Subsequently, the sections underwent an additional washing step before being covered with the original DAPI solution. After a final washing, the sections were mounted using an anti-fade reagent and examined under a fluorescence microscope (Nikon Eclipse Ti2, Tokyo, Japan). The excitation and emission wavelengths were as follows: DAPI—364/454 nm, Cy3—550/570 nm.

### 2.7. Transmission Electron Microscope (TEM)

Brain tissues were cut into 1 mm^3^ blocks and immediately fixed in 2.5% glutaraldehyde for 2 h. After rinsing three times with PBS for 15 min each, samples were fixed in 1% osmium tetroxide for 1.5 h. The tissues were dehydrated with 50%, 70%, and 90% ethanol for 15 min each, then placed in a 1:1 mix of 90% ethanol and acetone for 15 min, followed by 100% acetone for 10 min. They were treated with acetone and Epon812 mixtures (2:1 and 1:2) for 2 h and left overnight in pure resin. The following day, samples were embedded in fresh Epon812 resin, polymerized at 37 °C, and ultrathin-sectioned at 50–70 nm using an ultramicrotome. These were stained with 4% uranyl acetate and lead citrate for 15 min each. Finally, the ultrastructure was examined using a TEM (Hitachi HT7800, Tokyo, Japan).

### 2.8. 16S rDNA Sequencing

The extraction of total genomic DNA from mouse feces, PCR amplification, quantification of PCR products, pooling of samples for sequencing, and subsequent bioinformatics analysis were conducted with the support of Shanghai Origin-gene Biomedical Technology Co., Ltd. (Shanghai, China). The evaluation of PCR products to construct a qualified sequencing library was performed using the Agilent 2100 Bioanalyzer and the Illumina platform (Kapa Biosciences, Wilmington, MA, USA). Following denaturation into single strands, paired-end sequencing (2 × 250 bp) was executed using the NovaSeq 6000 sequencer (Illumina, San Diego, CA, USA). Microbial community analysis was performed using a standard bioinformatics workflow. Briefly, raw sequencing data underwent quality control, denoising with DADA2, diversity estimation, taxonomic classification, and statistical comparison between groups.

### 2.9. Cell Culture and Treatment

The HT22 cell line was sourced from Beyotime Biotechnology Co., Ltd. (Shanghai, China). The TFEB overexpression cell line was kindly provided by Professor ZhenXing Liu from Tianjin University of Science and Technology [23]. Following culture in complete DMEM (10% FBS, 1% penicillin-streptomycin) at 37 °C and 5% CO_2_, cells were exposed to 50, 100, and 200 μg/mL NiSO_4_. To inhibit ferroptosis or activate autophagy, cells were co-administered 200 μg/mL NiSO_4_ with either 1 μM ferrostatin-1 (Fer-1) or 2 μM Rapamycin (RAPA) for 24 h (MedChemExpress, Monmouth Junction, NJ, USA).

### 2.10. Analysis of Cytotoxicity via CCK-8 and LDH

After seeding HT22 cells (1 × 10^4^ cells/well) in 96-well plates overnight, the medium was replaced with NiSO_4_-containing medium (0–200 μg/mL) for 24 h. Cell viability was then assessed using the CCK-8 assay (SolarBio, Beijing, China) according to the instructions of manufacturer. Absorbance was measured at 450 nm after a 1 h incubation at 37 °C. The secretion amount of LDH was determined according to the concentration of extracellular LDH in damaged nerve cells by using the LDH enzyme-linked immunosorbent assay kit (SolarBio, Beijing, China). After treatment with NiSO_4_, the culture medium was used to detect the level of LDH according to the instructions in the kit manual.

### 2.11. RNA Sequencing Analysis

After 24 h of NiSO_4_ treatment, both control and experimental groups were trypsin-digested, centrifuged, and mixed with Trizol buffer on ice before being stored in liquid nitrogen. Upon revival, total RNA was extracted and quality-checked, followed by sequencing library construction using the ScriptSeq RNA Complete Kit. mRNA sequencing was conducted on the HiSeq 4000 from Origin-gene Company (Origin-gene, Shanghai, China).

### 2.12. Detection of Oxidative Stress

Intracellular ROS levels were detected using the fluorescent probe 2′,7′-dichlorodihydrofluorescein diacetate (DCFH-DA). After NiSO_4_ treatment, HT22 cells were washed twice with PBS. Cells were then incubated with 10 μM DCFH-DA (MedChemExpress, Monmouth Junction, NJ, USA) dissolved in KRBBS for 20 min at 37 °C in the dark. Following incubation, the DCFH-DA solution was aspirated, and cells were washed twice with PBS to remove any residual probe. Fluorescence was immediately visualized and captured using a fluorescence microscope (Nikon Eclipse Ti2, Tokyo, Japan). Images were acquired from at least five random fields per treatment group. For quantitative analysis, fluorescence intensity was measured using ImageJ software (version 1.53; National Institutes of Health, Bethesda, MD, USA) from three independent experiments. Oxidative stress indicators in HT22 cells, stimulated with NiSO_4_, were assessed using SolarBio Life Science kits to measure Superoxide dismutase (SOD), Catalase (CAT), Malondialdehyde (MDA), and GSH levels (SolarBio, Beijing, China).

### 2.13. Assessment of Mitochondrial Membrane Potential Using JC-1 Staining

Mitochondrial membrane potential was assessed using the JC-1 staining kit (AMRESCO, Solon, OH, USA) according to the manufacturer’s instructions. After NiSO_4_ treatment, cells cultured in 12-well plates were washed twice with PBS and incubated with JC-1 working solution (2 μM) for 30 min at 37 °C in the dark. Following incubation, cells were washed twice with JC-1 assay buffer to remove excess dye. Fluorescence was immediately visualized using a fluorescence microscope (Nikon Eclipse Ti2, Tokyo, Japan) with dual emission filters: 590 nm (red) for JC-1 aggregates and 530 nm (green) for JC-1 monomers. Fluorescence intensity was quantified using ImageJ software (version 1.53). The mitochondrial membrane potential was expressed as the ratio of red to green fluorescence intensity, with a decrease in this ratio indicating mitochondrial depolarization.

### 2.14. Iron (Fe^2+^) Fluorescent Probe

The working solution is prepared by using pre-warmed serum-free cell culture medium to formulate a RhoNox-1 (MedChemExpress, Monmouth Junction, NJ, USA) working solution with a concentration of 10 μM. Briefly, cells were seeded in a six-well plate. After adherence, the cells were incubated with 1000 μL of the dye working solution for 30 min at 37 °C, with gentle shaking to ensure complete coverage. The dye working solution was then carefully aspirated, and the cells were washed with fresh culture medium 2–3 times. Finally, the fluorescence was immediately observed and captured using a fluorescence microscope (Nikon Eclipse Ti2, Tokyo, Japan).

### 2.15. Western Blotting

For protein extraction, HT22 cells were harvested by scraping from 6-well plates, while brain tissues were dissected, flash-frozen in liquid nitrogen, and mechanically homogenized. Both cell and tissue samples were lysed in RIPA buffer supplemented with protease and phosphatase inhibitors, subjected to brief sonication on ice, and clarified by centrifugation at 12,000 rpm for 15 min at 4 °C to collect the supernatant. The protein solution was mixed with loading buffer, boiled for denaturation, and 40 μg from each group was separated using SDS-PAGE. Proteins were transferred to a nitrocellulose membrane, blocked, and incubated with primary antibodies overnight, followed by secondary antibodies for 60 min. Protein bands were visualized by incubating the membranes with an ECL substrate (Beyotime, Shanghai, China) and then imaging the chemiluminescent signals using Image Lab software (version 6.1; Bio-Rad Laboratories, Hercules, CA, USA). Antibodies of *GPX4*, *ACSL4*, heme oxygenase-1 (*HO-1*), nuclear factor erythroid 2-related factor 2 (*Nrf2*), *NCOA4*, *LC3*, zonula occludens-1 (*ZO-1*) and Occludin were supplied by Proteintech Group Inc. (Wuhan, China). Antibodies of *TFEB*, *p62*, *ATG5*, Unc-51-like autophagy protein 1 (*ULK1*), mechanistic target of rapamycinm (*mTOR*) and β-actin were purchase from Cell Signaling Technology Inc. (Danvers, MA, USA).

### 2.16. Statistical Analysis

Data from three independent experiments, presented as mean ± SD, were analyzed for statistical significance using one-way ANOVA with Tukey’s post hoc test in GraphPad Prism 8.0. A *p*-value below 0.05 was considered statistically significant.

## 3. Results

### 3.1. NiSO_4_-Induced Brain Injury Through Ferroptosis

**Figure 1:**
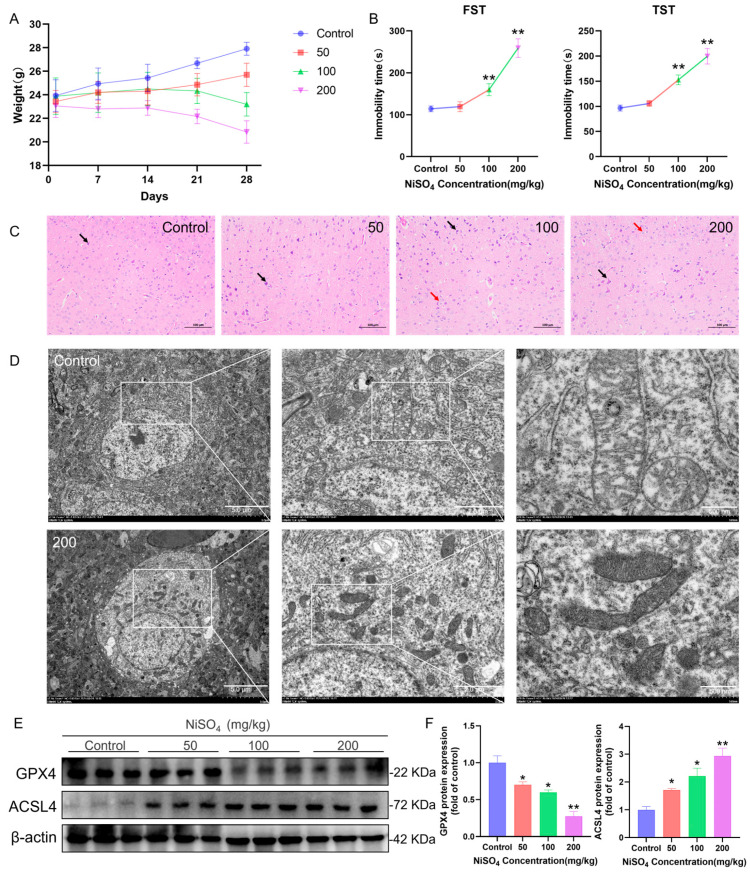
NiSO_4_-induced brain injury through ferroptosis. (**A**) Changes in body weight of mice (n = 6 mice per group). (**B**) Tail suspension test (TST) and forced swim test (FST) behavioral assessments (n = 6 mice per group). (**C**) Histological examination of brain tissues using hematoxylin and eosin (H&E) staining, black arrows indicated neuronal shrinkage, and red arrows indicated neuronal degeneration (scale bar = 100 μm). (**D**) Mitochondrial morphology in mouse brain tissues visualized by transmission electron microscopy (scale bar = 2 μm). (**E**,**F**) Western blot analysis of ACSL4 and GPX4 protein expressions. Data were quantified using ImageJ software (n = 3 independent experiments). All data are presented as mean ± SD. Statistical significance was determined by one-way ANOVA followed by Tukey’s post hoc test. Statistical significance versus the control group is indicated as * *p* < 0.05 and ** *p* < 0.01.

Following 28-day gastric gavage in mice, alterations in body weight was depicted in Figure 1A. The administration of NiSO_4_ significantly influenced the body weight gain of the mice. Behavioral assessments, including the TST and FST, showed a marked increase in immobility time in NiSO_4_-treated mice compared with controls, indicating the induction of depressive-like behaviors (Figure 1B). Histopathological examination of the mouse brain revealed dose-dependent changes associated with NiSO_4_ exposure. As depicted in Figure 1C, the high-dose group exhibited increased neuronal shrinkage (indicated by black arrows) in the cerebral cortex, with intensified cell staining and an indistinct nuclear-cytoplasmic boundary; a subset of neurons displayed degeneration (indicated by red arrows), characterized by cytoplasmic vacuolization. TEM analysis demonstrated that in the high dose group, mitochondria appeared shrunken with increased membrane density, outer membrane rupture, and reduction or loss of cristae—ultrastructural features consistent with ferroptosis (Figure 1D). Furthermore, Western blot analysis revealed a significant decrease in GPX4 and a concomitant increase in ACSL4 (Figure 1E,F), supporting the conclusion that NiSO_4_ triggers ferroptosis in the brain.

### 3.2. NiSO_4_-Induced Gut Microbiota Dysbiosis

**Figure 2:**
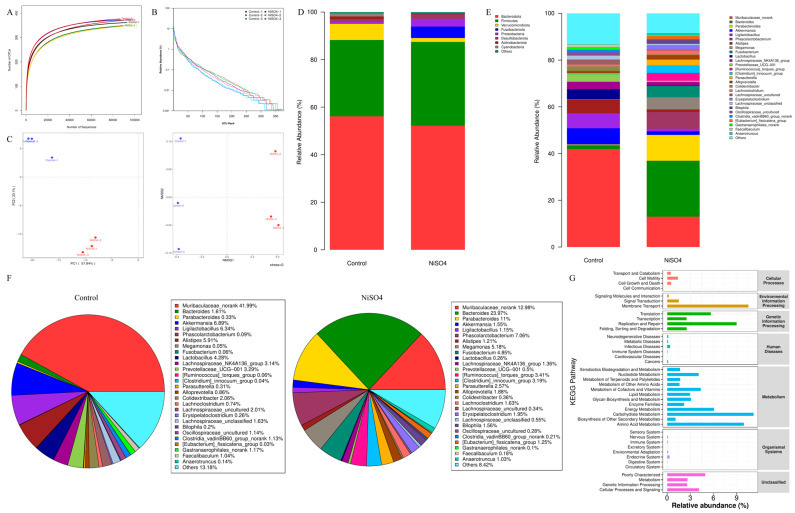
NiSO_4_-induced gut microbiota dysbiosis. (**A**) Species rarefaction curve. (**B**) Abundance rank curve. (**C**) PCA analysis, NMDS analysis. (**D**) Composition of the gut microbiota at the phylum level in control and NiSO_4_-treated mice. (**E**) Composition of the gut microbiota at the genus level in control and NiSO_4_-treated mice. (**F**) Genus-level composition of the gut microbiota. (**G**) KEGG metabolic pathway difference analysis diagram.

After processing the raw data from 16S rDNA sequencing, the species rarefaction curve’s gradual saturation indicated that the sequencing depth was sufficient to meet detection requirements (Figure 2A). In comparison to the NiSO_4_ group, the abundance rank curve of the control group was more leveled and exhibited a greater horizontal span (Figure 2B), indicating that the control group possessed greater homogeneity and species richness than the NiSO_4_ group. The β-diversity and NMDS analyses corroborated that the intestinal microbial composition in the NiSO_4_-treated group exhibited significant divergence from that of the control group (Figure 2C). At the phylum level, NiSO_4_ exposure notably modified the intestinal microbiota composition compared to the control group, as evidenced by a marked decrease in Verrucomicrobia, Bacteroidota, Cyanobacteria, and Actinobacteria, coupled with an increase in Fusobacteriota, Firmicutes, Proteobacteria, and Desulfobacterota (Figure 2D). At the genus level, the NiSO_4_ group demonstrated a downward trend in the relative abundances of Muribaculaceae_norank, Akkermansia, Ligilactobacillus, Alistipes, and Lactobacillus. Conversely, there was an observed increase in the relative abundances of Bacteroides, Parabacteroides, Phascolarctobacterium, Megamonas, and Fusobacterium in the NiSO_4_ group (Figure 2E). Furthermore, analysis of the top five relative abundances further confirmed that NiSO_4_ exposure altered the microbial proportions observed in the control group, specifically resulting in a decreased proportion of Muribaculaceae_norank and an increased proportion of Bacteroides. Furthermore, within the Bacteroidetes phylum, Parabacteroides emerged as the most prominent feature in the NiSO_4_ group, whereas Akkermansia exhibited the highest prevalence in the control group (Figure 2F). Analysis of Figure 2D,F indicated that the phyla Verrucomicrobia, Bacteroidota, Fusobacteriota, and Firmicutes demonstrated the most pronounced variation trends. The consumption of NiSO_4_ was observed to promote the proliferation of pathogenic bacteria, such as Erysipelatoclostridium within the Clostridium genus, while diminishing the presence of beneficial microbial populations, including Ligilactobacillus and Colidextribacter. KEGG pathway enrichment analysis of intestinal microbiota revealed significant associations with pathways implicated in neurodegenerative disorders and immune-system dysregulation (Figure 2G).

### 3.3. NiSO_4_ Compromises Intestinal Barrier Integrity and Activates Gut–Brain Axis

**Figure 3:**
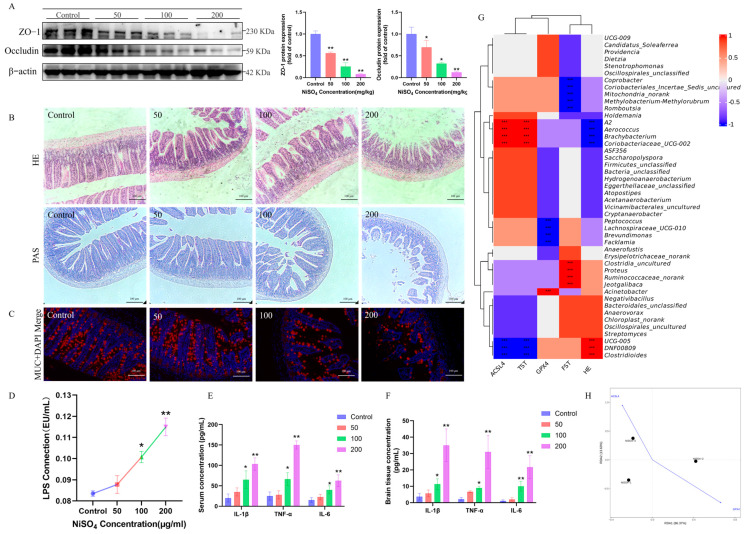
NiSO_4_ compromises intestinal barrier integrity and activates gut-brain axis. (**A**) Expressions of *ZO-1* and *Occludin*. (**B**) HE and PAS staining. (**C**) Expression of *MUC2*. (**D**) Serum LPS content. (**E**) *IL-1β*, *TNF-α*, and *IL-6* in serum (n = 6). (**F**) *IL-1β*, *TNF-α*, and *IL-6* in brain tissues (n = 6). (**G**) Gut–brain correlation heatmap. (**H**) RDA analysis. All data are presented as mean ± SD. Statistical significance was determined by one-way ANOVA followed by Tukey’s post hoc test. Statistical significance versus the control group is indicated as * *p* < 0.05, ** *p* < 0.01, *** *p* < 0.001.

An examination of intestinal barrier integrity was performed, NiSO_4_ treatment significantly impaired the expression of the tight junction proteins *ZO-1* and Occludin in the ileum (Figure 3A). Histological examination of HE and PAS-stained ileum sections indicated a thinning of the intestinal wall and a reduction in villus height in the NiSO_4_ group. Goblet cells are responsible for secreting mucin, which contributes to the formation of a crucial mucus barrier within the intestine. However, exposure to NiSO_4_ induced a significant decrease in the goblet cell population in the intestinal epithelium, as illustrated in Figure 3B. The ileal immunofluorescence results, depicted in Figure 3C, further corroborated that NiSO_4_ significantly downregulated the expression of the MUC2 protein. Additionally, the serum LPS content demonstrated a dose-dependent increase, as shown in Figure 3D. These findings suggest that NiSO_4_-induced intestinal injury compromises the integrity of the intestinal barrier. Furthermore, NiSO_4_ induced a marked increase in the concentrations of *IL-1β*, *TNF-α*, and *IL-6* in serum and brain tissues, with the most pronounced increase observed in the high-dose group (Figure 3E,F). Correlation analysis integrating 16S rDNA sequencing data of the intestinal microbiota with brain injury-related parameters revealed a significant negative association between specific microbial profiles and brain GPX4 levels, alongside a positive correlation with ACSL4 expression (Figure 3H). As illustrated in Figure 3G, the presence of Clostridioides, DNF00809, and UCG-005 in the murine intestine exhibits a positive correlation with HE injury in the murine brain. Similarly, Aerococcus, Brachybacterium, and Coriobacteriaceae UCG-002 demonstrated a positive correlation with the brain ferroptosis-associated proteins *ACSL4* and TST. Conversely, Peptococcus, Lachnospiraceae UCG-010, Brevundimonas, and Facklamia were negatively correlated with the brain ferroptosis protein *GPX4*. Additionally, Clostridia uncultured, Proteus, Ruminococcaceae_norank, and Jeotgalibaca showed a positive correlation with FST, whereas Coprobacter, Coriobacteriales Incertae Sedis unci, Ured, Mitochondria norank, Methylobacterium-Methylorubrum, and Romboutsia were negatively correlated with FST. These findings suggested that an increase in certain detrimental intestinal bacteria was associated with brain injury, potentially through dysbiosis-induced activation of the gut–brain axis.

### 3.4. NiSO_4_-Induced Cytotoxicity and Transcriptome Analysis in HT22 Cells

**Figure 4:**
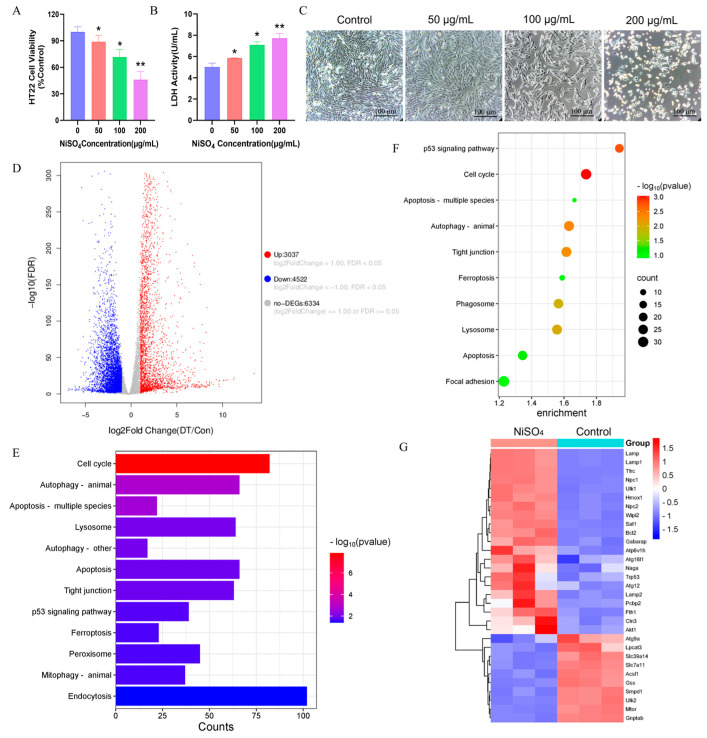
NiSO_4_-induced cytotoxicity and transcriptome analysis in HT22 cells. (**A**) Cell viability of HT22 cells was determined by the CCK-8 assay. (**B**) The LDH release level. (**C**) After treating HT22 cells with NiSO_4_ (0–200 μg/mL) for 24 h, the morphology was observed using an inverted microscope (scale bar = 100 μm). (**D**) Volcano plot of DEGs identified by RNA-seq (n = 3). (**E**) Bar chart of KEGG pathways significantly enriched in differentially expressed transcripts. (**F**) A KEGG pathway enrichment bubble chart highlighting significantly upregulated EDGs. (**G**) Clustering diagram of expression patterns of significantly differentially expressed genes. All data are presented as mean ± SD. Statistical significance was determined by one-way ANOVA followed by Tukey’s post hoc test. Statistical significance versus the control group is indicated as * *p* < 0.05 and ** *p* < 0.01.

To assess the cytotoxic effects of NiSO_4_, the absorbance of CCK-8 was recorded, and LDH activity was measured. As illustrated in Figure 4A–C, NiSO_4_ elicited a dose-dependent reduction in cell viability. Concurrently, LDH activity in the culture medium exhibited a progressive increase with escalating doses. Microscopic examination revealed a decrease in cell density, indicative of heightened cell mortality. The distribution of differentially expressed genes (DEGs) in HT22 cells following exposure to 200 μg/mL NiSO_4_ was analyzed. As depicted in Figure 4D, NiSO_4_ significantly modulated the expression of 7559 genes, with 3037 genes being upregulated and 4522 genes downregulated. KEGG pathway analysis revealed significant enrichment of the differentially expressed genes in key pathways, including autophagy, lysosome, ferroptosis, apoptosis, and the P53 signaling pathway (Figure 4E). The group treated with NiSO_4_ exhibited a significant upregulation of pathways associated with ferroptosis, autophagy, lysosome, and apoptosis (Figure 4F). Additionally, the expression levels of key genes within these pathways were markedly altered (Figure 4G). These findings suggested that the toxicological mechanism of NiSO_4_ may involve processes related to ferroptosis, autophagy, and lysosomal activity.

### 3.5. NiSO_4_ Triggers Neurotoxicity via Oxidative Stress—Mediated Ferroptosis

**Figure 5:**
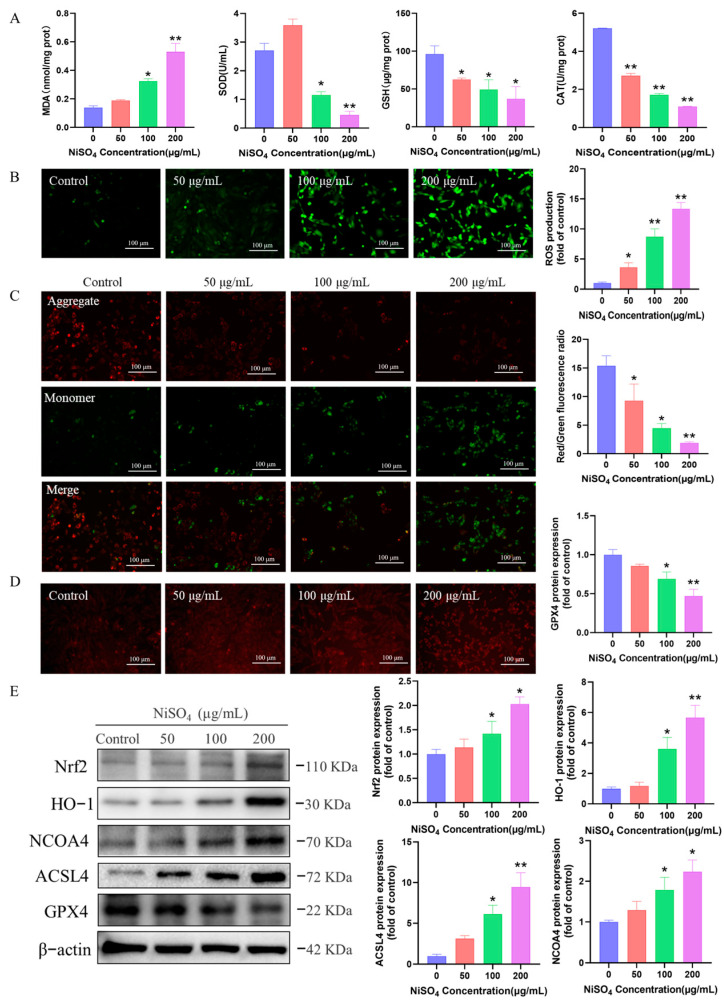
NiSO_4_ triggers neurotoxicity via oxidative stress-mediated ferroptosis. (**A**) Oxidative stress parameters including SOD and CAT activities, and GSH and MDA levels (n = 3). (**B**) Intracellular ROS detection using DCFH-DA probe (10 μM, 20 min incubation) analyzed by fluorescence microscopy (scale bar = 100 μm). (**C**) Mitochondrial membrane potential assessed by JC-1 staining (2 μM, 30 min incubation; scale bar = 100 μm). (**D**) Intracellular Fe^2+^ levels detected using RhoNox-1 probe (10 μM, 30 min incubation; scale bar = 100 μm). (**E**) Western blot analysis of *HO-1*, *Nrf2*, *NCOA4*, *ACSL4* and *GPX4* protein expressions (n = 3 independent experiments). All data are presented as mean ± SD. Statistical significance was determined by one-way ANOVA followed by Tukey’s post hoc test. Statistical significance versus the control group is indicated as * *p* < 0.05 and ** *p* < 0.01.

In the context of antioxidant markers, CAT, GSH, and SOD were significantly downregulated, while MDA levels were markedly elevated following NiSO_4_ exposure (Figure 5A). The DCFH-DA assay indicated a significant upregulation of reactive oxygen species (ROS) in HT22 cells upon NiSO_4_ stimulation. The observed enhancement in green fluorescence suggested that NiSO_4_ facilitated in a dose-dependent increase in ROS accumulation (Figure 5B). Furthermore, JC-1 staining results demonstrated that mitochondrial damage intensified with increasing doses of NiSO_4_ (Figure 5C). Additionally, as shown in Figure 5E, *Nrf2* and *HO-1*, proteins associated with the antioxidant pathway, were significantly upregulated. In contrast, levels of the ferroptosis-related proteins ACSL4 and NCOA4 were markedly increased, while GPX4 expression was substantially downregulated. The measurement of Fe^2+^ probe content revealed an increase correlating with higher doses (Figure 5D). These findings suggested that NiSO_4_-induced ferroptosis by initiating oxidative stress, thereby impairing the antioxidant defense mechanisms.

### 3.6. NiSO_4_ Activates the Autophagic-Lysosomal Pathway in HT22 Cells

**Figure 6:**
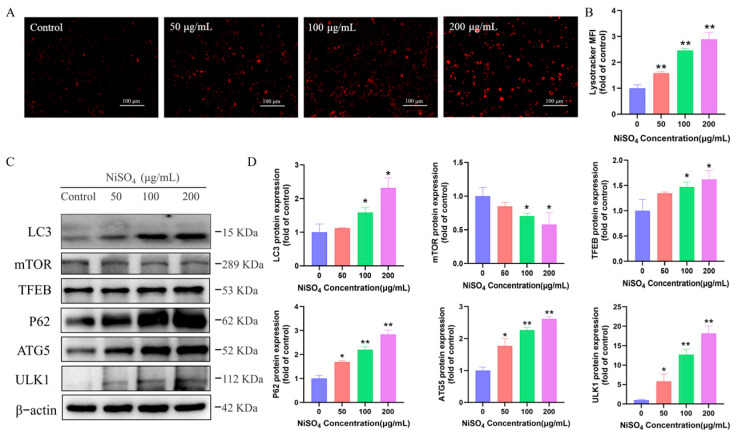
NiSO_4_ activates the autophagic-lysosomal pathway in HT22 cells. (**A**,**B**) Lysosomes were labeled with the Lysotracker Red fluorescent probe (Scale bar = 100 μm). (**C**,**D**) Protein expressions of *mTOR*, *LC3*, and *TFEB*, *p62*, *AGT5*, and *ULK1* were detected by Western blotting (n = 3 independent experiments). Data were analyzed with ImageJ. All data are presented as mean ± SD. Statistical significance was determined by one-way ANOVA followed by Tukey’s post hoc test. Statistical significance versus the control group is indicated as * *p* < 0.05 and ** *p* < 0.01.

Through transcriptome analysis, it was hypothesized that NiSO_4_-induced damage to HT22 cells may be associated with autophagy. Lysosomal content was assessed using the LysoTracker Red fluorescent probe, revealing a dos-dependent increase in lysosomal abundance (Figure 6A,B). Western blot analysis further confirmed significant upregulation of the autophagy markers LC3-II and p62 (Figure 6C,D). Furthermore, NiSO_4_ treatment resulted in the overexpression of key autophagy regulators, including *ULK1*, *TFEB*, and *ATG5*, while *mTOR* expression exhibited an inverse trend. These findings collectively suggest that NiSO_4_ activates the autophagic-lysosomal signaling pathway in HT22 cells.

### 3.7. Fer-1 Attenuates NiSO_4_-Induced Neurotoxicity and Suppresses Autophagic Activation

**Figure 7:**
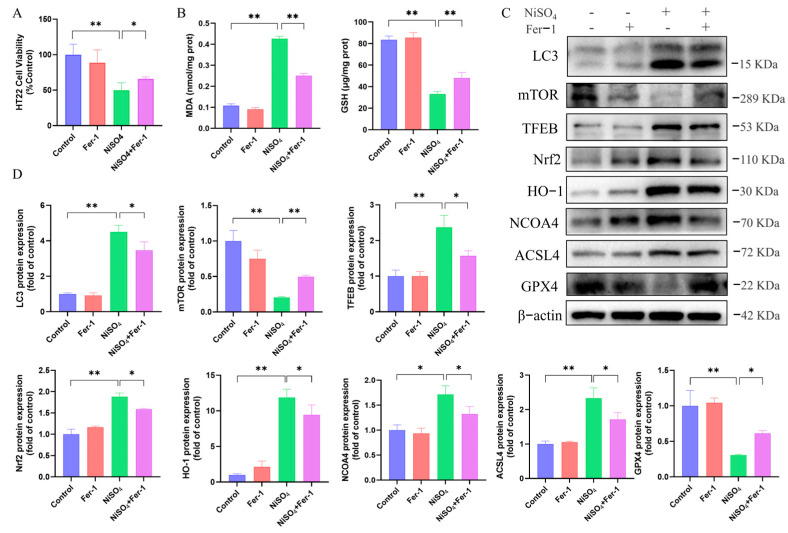
Fer-1 attenuates NiSO_4_-induced neurotoxicity and suppresses autophagic activation (**A**) Effect of Fer-1 on NiSO_4_-induced cell viability. (**B**) Effects of Fer-1 on the levels of MDA and GSH. (**C**,**D**) Protein expressions related to autophagy and ferroptosis were detected by Western blotting (n = 3 independent experiments). Data were analyzed with ImageJ. All data are presented as mean ± SD. Statistical significance was determined by one-way ANOVA followed by Tukey’s post hoc test. Statistical significance versus the control group is indicated as * *p* < 0.05 and ** *p* < 0.01.

Fer-1 was employed to further elucidate the role of ferroptosis in NiSO_4_-induced neurotoxicity. Fer-1 functions as a ferroptosis inhibitor by impeding lipid peroxidation through the inhibition of cystine transport and glutathione synthesis. As demonstrated in Figure 7A, the application of Fer-1 resulted in enhanced cell viability. Pretreatment with Fer-1 was associated with decrease in MDA levels and an elevation in GSH levels (Figure 7B). Figure 7C,D revealed that, the Fer-1 treatment group exhibited a marked reduction in levels of *HO-1*, *Nrf2*, *ACSL4*, and *NCOA4* proteins, alongside an increase in *GPX4* protein expression. Concurrently, the Fer-1 treatment suppressed the levels of *LC3* and *TFEB* while conversely elevating *mTOR* expression. These findings suggested that the inhibition of ferroptosis is concomitant with a reduction in autophagy, implying that autophagy may serve a protective function in the context of ferroptosis. However, the protection was partial, suggesting that ferroptosis is an important, but not the exclusive, cell death pathway activated by NiSO_4_ in HT22 cells.

### 3.8. TFEB-Driven Autophagy—Lysosomal Pathway Mediates Protection Against NiSO_4_-Induced Ferroptosis

**Figure 8:**
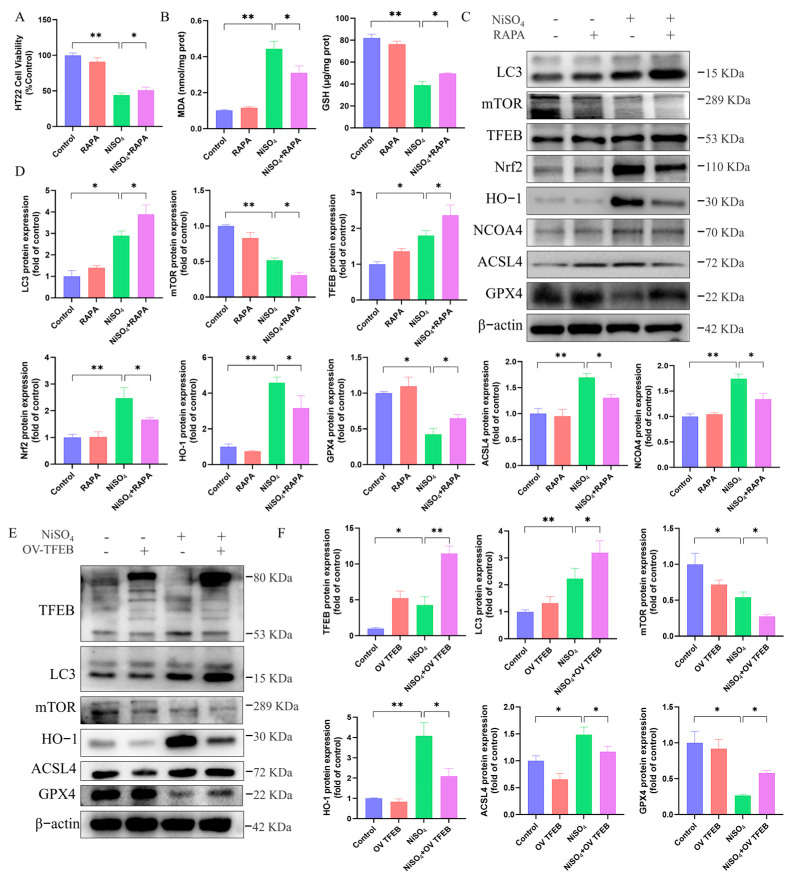
TFEB-driven autophagy–lysosomal pathway mediates protection against NiSO_4_-induced ferroptosis. (**A**) Effect of RAPA on NiSO_4_-induced cell viability. (**B**) Effect of RAPA on GSH and MDA levels. (**C**,**D**) Protein expressions related to autophagy and ferroptosis were detected by Western blotting. (**E**,**F**) Effect of *TFEB* on NiSO_4_-induced protein expressions associated with autophagy and ferroptosis (n = 3 independent experiments). All data are presented as mean ± SD. Statistical significance was determined by one-way ANOVA followed by Tukey’s post hoc test. Statistical significance versus the control group is indicated as * *p* < 0.05 and ** *p* < 0.01.

To further elucidate the relationship between autophagy and ferroptosis, autophagy was activated using RAPA, which is known to inhibit the *mTOR* pathway, thereby inducing and promoting autophagy. As illustrated in Figure 8A, treatment with the RAPA significantly improved cell viability in the presence of NiSO_4_, yet the rescue effect was incomplete. This indicates that while the augmentation of autophagy confers protection, other cell death mechanisms contribute substantially to the overall toxicity. Furthermore, the treatment significantly enhanced the antioxidant capacity, demonstrated by a decrease in MDA and an upregulation in GSH levels (Figure 8B). Figure 8C,D demonstrated that RAPA treatment facilitated autophagy, as evidenced by corresponding changes in protein expression. Specifically, there was an increase in *GPX4* and *NCOA4* levels and a decrease in *ACSL4* expression. Our results supported the notion that autophagy served as a protective mechanism to ameliorate ferroptosis triggered by NiSO_4_ exposure. To further investigated the crosstalk between autophagy–lysosome and ferroptosis, *TFEB* overexpressed cell line was used. Figure 8E,F demonstrated that *TFEB* overexpression shifts the molecular profile away from ferroptosis, evidenced by decreased *ACSL4*/*HO-1* and increased *GPX4* expression. These results indicated that the autophagy–lysosome pathway functioned as a key mechanism through which TFEB conferred protection against NiSO_4_-induced ferroptosis.

## 4. Discussion

Nickel is a metal that is ubiquitously present in the environment and has emerged as a significant pollutant due to its extensive use in industrial applications. Growing evidence has demonstrated the adverse toxicological effects of nickel exposure [24]. Known mechanisms of nickel-induced toxicity include oxidative stress, apoptosis, autophagy, and cell cycle arrest [25,26,27]. Ferroptosis, first characterized by Stockwell and colleagues in 2012, is an iron-dependent form of regulated cell death marked by the accumulation of lipid peroxides and distinct from apoptosis [28]. Emerging studies have implicated ferroptosis in the pathogenesis of various diseases, including neurological disorders, cardiovascular conditions, and metabolic syndromes [29,30,31]. However, the involvement of NiSO_4_ in brain injury through ferroptosis remains unclear. In this study, we evaluated ferroptosis indicators both in vivo and in vitro following NiSO_4_ exposure.

Ferroptosis is initiated by oxidative disturbances within the intracellular microenvironment, which is regulated by *GPX4*. The presence of Fe^2+^ and lipid peroxidation are critical factors in the induction of ferroptosis [32]. *ACSL4* has been identified as a significant contributor to the execution of ferroptosis by facilitating the esterification of arachidonic acid (AA) or adrenic acid (AdA) into phosphatidylethanolamine (PE) [33]. In the present study, a decrease in *GPX4* protein expression and an increase in *ACSL4* expression were observed, identifying these changes as biomarkers of ferroptosis. Ferroptosis was closely linked to mitochondrial function, with evidence suggesting that mitochondrial dysfunction and structural damage exacerbated oxidative stress, thereby driving ferroptotic cell death [34]. Our study further demonstrated mitochondrial atrophy in the mouse brain through ultrastructural analysis, alongside dose-dependent alterations in the levels of GSH, SOD, MDA, CAT, and lipid ROS in HT22 cells. These findings corroborated the induction of ferroptosis in the mouse brain and HT22 cells by NiSO_4_ exposure. Additionally, it has been reported that treatment with Fer-1 alleviated the cytotoxicity of nickel-induced damage in TCMK-1 cells [16]. Similarly, our cell viability assays demonstrated that treatment with Fer-1 mitigated the cytotoxic effects in nickel-induced HT22 cells, further supporting the role of ferroptosis as a key contributor to NiSO_4_-induced cell death. Previous research has established that nickel-induced autophagy in the mouse brain [26]. Consistent with this, our RNA sequencing analysis and experimental validation confirmed that NiSO_4_ triggered autophagy in mouse hippocampal neurons. Autophagy serves as a fundamental process that delivers cellular components to lysosomes for degradation and recycling. Accumulating evidence suggested that autophagy exerted a protective role in various pathological contexts [11]. However, excessive autophagy may facilitate ferroptosis. The ablation of key autophagy or lysosome-dependent cell death effectors, such as *STAT3* and cathepsin B, could inhibit ferroptotic cancer cell death, whereas the knockout of *SLC7A11* or *GPX4* could reduce autophagy, thereby providing further protection against ferroptosis induced by Golgi stress. Moreover, RAPA has been shown to induce pro-survival autophagy at low doses, but at higher doses, it could trigger autophagy-dependent ferroptosis. These findings suggested a complex interplay among autophagy, ferroptosis, and organelle stress [35]. To elucidate the relationship between autophagy and NiSO_4_-induced ferroptosis, the ferroptosis inhibitor Fer-1 was employed in this study. The results demonstrated a concomitant decrease in autophagy expression following ferroptosis inhibition, indicating a linkage between autophagy and ferroptosis. To further investigate the mechanisms underlying NiSO_4_-induced ferroptosis and autophagy, the autophagy activator RAPA was utilized. The experimental outcomes revealed that, in comparison to NiSO_4_ treatment alone, RAPA effectively suppressed ferroptosis formation. Our pharmacological intervention data revealed that Fer-1 or RAPA significantly rescued cell viability, the effect was partial. This indicates that NiSO_4_ triggers a multimodal cell death response in HT22 neurons. Ferroptosis and regulated autophagy are important components of this response, but other parallel pathways, such as apoptosis, necroptosis, or unregulated necrosis, likely contribute to the final toxic outcome. The incomplete rescue further underscores the complexity of nickel’s neurotoxic mechanism and suggests that effective neuroprotection might require a combinatorial strategy targeting multiple cell death pathways simultaneously. Future studies are warranted to delineate the full spectrum of death modalities activated by nickel. Nevertheless, the present findings indicated that autophagy played a protective role in reducing the incidence of ferroptosis. To elucidated the function of the *mTOR*-*TFEB* pathway in NiSO_4_-induced ferroptosis, we performed a comparative analysis using Hela cells with stable *TFEB* overexpression and wild-type controls. The results demonstrated that *TFEB*-overexpressing cells exhibited significantly reduced expression of key ferroptosis-related proteins compared to control cells, indicating that *TFEB* activation negatively regulated ferroptosis induced by NiSO_4_.

Alterations in the microbiota-gut–brain axis, a bidirectional communication network linking the central nervous system and the gastrointestinal tract, have been implicated in the pathogenesis of various mental disorders [36]. Accumulating evidence has elucidated the role of the microbiota-gut–brain axis in neurodevelopmental disorders, highlighting the physiological and pathophysiological mechanisms influenced by the gut microbiota in these conditions [37]. Notably, nickel exposure has been shown to induce gut microbial dysbiosis, underscoring a well-established link between neurological diseases and gut microbiota composition [38]. Increasing evidence suggested that dysbiosis, characterized by alterations in the bacterial composition of the gut microbiota, could compromise host immune homeostasis and intestinal barrier integrity. Such dysbiosis fostered a pro-inflammatory environment and systemic endotoxemia, thereby contributing to the pathogenesis of neurodegenerative diseases and metabolic disorders [39]. Research has indicated that the bacterial diversity in the fecal matter of mice experiencing severe stroke was diminished, whereas the microbiome of mice with mild stroke remained comparable to that of the control group. These overall alterations were characterized by a reduction in Firmicutes and a concurrent proliferation of Bacteroidetes, which constituted the two predominant bacterial phyla in the intestine. This microbial shift was correlated with decreased intestinal peristalsis and increased permeability of the intestinal wall [40]. However, upon examining microbiota changes at more specific taxonomic levels, some studies have identified distinct bacterial alterations, such as an increase in the Firmicutes phylum, a decrease in Prevotellaceae, and an elevated abundance of Firmicutes [41]. These previously reported patterns were consistent with the microbial changes observed in our study at both phylum and genus levels, suggesting that NiSO_4_ may influence brain function through gut microbiota modulation. In addition, LPS, a pathogen-associated molecular pattern derived from the outer membrane of Gram-negative bacteria, may contribute to this process as a potent immunostimulatory endotoxin [42]. The observed reduction in Akkermansia and Lactobacillus was particularly significant. These genera were pivotal producers of short-chain fatty acids (SCFAs), such as butyrate and acetate, which were crucial for maintaining intestinal barrier integrity and exerting systemic anti-inflammatory effects [43,44]. More recently, SCFAs, especially butyrate, have been implicated in the direct suppression of ferroptosis by upregulating the expression of GPX4 and other antioxidant defense molecules [45]. Therefore, their depletion in our model may not only weaken the intestinal barrier but also directly remove a key endogenous brake on ferroptosis in the brain. Conversely, the expansion of pro-inflammatory genera like Bacteroides and Parabacteroides could contribute to a state of persistent systemic inflammation, elevating circulating levels of pro-inflammatory cytokines such as TNF-α and IL-6, which were known to sensitize cells to ferroptosis [46]. Two critical pathophysiological indicators of neurodegenerative diseases are oxidative/nitrative stress and inflammation, which may be precipitated by enhanced intestinal permeability and an associated increase in pathogenic factors. These alterations facilitate the excessive release of LPS and other bacterial products into the circulatory system, thereby provoking chronic systemic inflammation and subsequently compromising the integrity of the blood–brain barrier (BBB). The compromised BBB permits the infiltration of potentially harmful bacterial products, including LPS, as well as activated neutrophils and leukocytes into the brain, culminating in neuroinflammation and apoptosis [47]. In our experimental model, we observed a significant increase in serum LPS levels in mice following exposure to NiSO_4_, supporting the role of gut-derived endotoxemia in the observed neurotoxicity.

Despite the insights provided by our study, several intriguing questions remain for future investigation. Firstly, while the HT22 cell line served as a robust model for dissecting the core mechanisms of NiSO_4_-induced ferroptosis, validating these findings in additional neuronal cell lines, particularly of human origin, would strengthen the generalizability of our conclusions and is a key direction for our subsequent work. More importantly, the precise causal relationship between specific gut microbial alterations and the initiation of ferroptosis in distant neurons warrants deeper exploration. Future studies employing sophisticated approaches, such as fecal microbiota transplantation, gnotobiotic animal models, or direct treatment of neuronal cultures with microbiota-derived metabolites, will be essential to move beyond correlation and definitively identify the bacterial components or signaling molecules that trigger the gut–brain–ferroptosis axis. Elucidating these detailed mechanisms will not only enhance our understanding of nickel neurotoxicity but may also reveal novel therapeutic targets for heavy metal-associated neurological disorders. Furthermore, it is important to consider the bioavailability of orally administered NiSO_4_. The solubility of Ni^2+^ is pH-dependent, and precipitation in the higher pH environment of the intestine likely limits its full absorption. Nevertheless, our data clearly demonstrate a dose-dependent neurotoxic effect, indicating that the fraction of nickel that remains bioavailable is sufficient to traverse the intestinal barrier, enter systemic circulation, and subsequently induce pathological changes in the brain. Future investigations will directly quantify nickel accumulation in the brain and other tissues following exposure to precisely correlate the administered dose with tissue burden and the observed toxicological outcomes.

## 5. Conclusions

In summary, our findings demonstrated that NiSO_4_-induced ferroptosis both in vivo and in vitro. Our data suggested that nickel ingestion may compromise intestinal barrier integrity, with gut microbiota dysbiosis showing a significant correlation with cerebral damage markers. Furthermore, our results underscored the involvement of autophagy–lysosome, mediated through the *mTOR*-*TFEB* pathway, in regulating ferroptotic responses. Collectively, this study not only delineated the novel crosstalk between autophagy and ferroptosis but also identified *TFEB* activation as a potential therapeutic avenue against nickel-related neuronal damage.

## Data Availability

The raw data supporting the conclusions of this article will be made available by the authors on request.

## Abbreviations

The following abbreviations are used in this manuscript:

ACSL4	Acyl-CoA Synthetase Long Chain Family Member 4
CAT	Catalase
CCK-8	Cell Counting Kit-8
DCFH-DA	2′7′-Dichlorofluorescin diacetate
DMEM	Dulbecco’s Modified Eagle Medium
FTH1	Ferritin Heavy Chain 1
FTL	Ferritin Light Chain
GPX4	Glutathione Peroxidase 4
GR	Glutathione Reductase
GST	Glutathione S-Transferase
GSH	Glutathione
GPx	Glutathione Peroxidase
HO-1	Heme oxygenase-1
LPS	Lipopolysaccharide
LPO	Lipid Peroxidation
mTOR	Mechanistic Target of Rapamycin
MUC2	Mucin 2
TFR1	Transferrin Receptor 1
MDA	Malondialdehyde
NCOA4	Nuclear Receptor Coactivator 4
Nrf2	Nuclear factor erythroid 2-related factor 2
RAPA	Rapamycin
ROS	Reactive oxygen species
SOD	Superoxide dismutase
TEM	Transmission Electron Microscopy
TFEB	Transcription Factor EB
ULK1	Unc-51-like autophagy protein 1
